# Okara protein extracted by alternating ultrasonic/alkali treatment and its improved physicochemical and functional properties

**DOI:** 10.1016/j.ultsonch.2024.107129

**Published:** 2024-10-28

**Authors:** Lu Tang, Xiaolin Liu, Shiru Bai, Dan Zhao, Xuzhen Guo, Dandan Zhu, Guiying Su, Bei Fan, Bo Wang, Liang Zhang, Fengzhong Wang

**Affiliations:** Institute of Food Science and Technology, Chinese Academy of Agricultural Sciences, Beijing 100193, China

**Keywords:** Okara, Protein, Alkali, Ultrasound, Functional properties, Emulsion stability

## Abstract

Okara protein (OP) is a potential plant-based protein that is beneficial to human health. In this work, an alternating ultrasonic/alkali treatment method with non-continued cavitation and thermal energy output was used to extract protein (AUA-OP) from okara to enhance the functional properties of OP and improve the stability of OP-based emulsions. The purity of AUA-OP was greater than 80%. Compared with traditional (physical-assisted) alkali treatment, FTIR and SDS-PAGE revealed that AUA-OP retained the chemical structure of the protein, but the number of ultrasound-induced exposure sites increased, with increased fluorescence intensity, surface hydrophobicity, and absolute ζ-potential. After alternating ultrasonic/alkali treatment, the protein particles were looser and smaller. In addition, the water/oil holding capacity, EAI, and ESI of AUA-OP further increased. The viscosity of the AUA-OP-stabilized emulsion was also greater. Finally, a 28-day emulsion storage assay revealed that the AUA-OP-stabilized emulsion was stable with a relatively low droplet size and creaming index, indicating great potential for the development of stable protein-based emulsions.

## Graphical abstract

**Figure:**
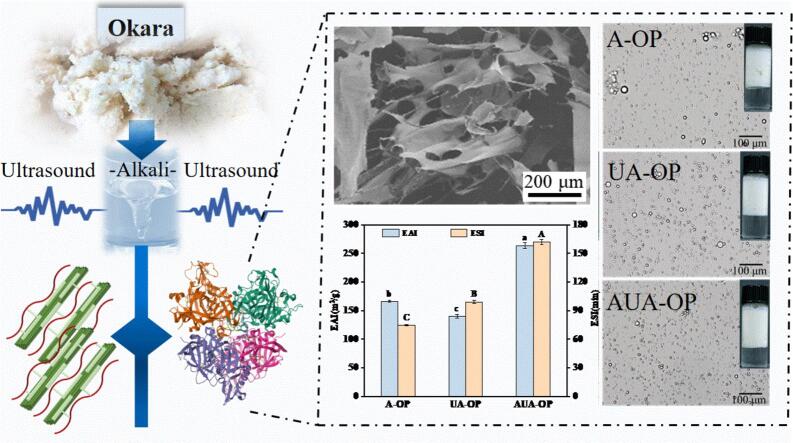

## 1 Introduction

Okara, as a byproduct after the deep processing of soybeans, is rich in dietary fiber (>65 %), protein (>20 %), and fat (>5%), indicating extremely high nutritional value [1], [2]. Currently, owing to the flourishing development of soybean products in the food industry, more than 20 million tons of okara, which are used mainly as animal feed, fermentation raw materials, or be landfilled, have accumulated annually, thus causing serious resource waste [3]. To date, the high-value utilization of okara has been in the development stage, and the extraction of active constituents from okara is an effective way to solve this problem [4].

In recent years, to address the fact that parts of the human body are intolerant and allergic to animal protein, the demand for plant-based proteins has increased [5], [6]. Okara protein (OP) is a functional substance with high activity, and its content in okara is second only to that of dietary fiber [4]. Research has revealed that OP has an essential amino acid profile, high bioavailability, and nonallergic behavior [7]. Compared with some high-quality plant-based proteins, OP shows similar functional properties, such as good emulsifying stability, foaming capability, and solubility [6]. As proteins with amphipathic properties can reduce the interfacial tension at the oil–water interface to improve the stability of protein-based emulsions, the emulsifying properties of proteins are highly important for food processing [8].

However, as traditional emulsifiers, proteins are often affected by their natural structure, processing methods, and storage time, leading to a decrease in their functional properties [9], [10], [11]. The extraction method is an important factor affecting protein properties. Alkali treatment is a typical pathway to extract proteins, in which proteins in okara can be dissolved in an alkaline solution to separate insoluble substances and then precipitated by adjusting the isoelectric point at pH 4.5 to obtain OP [6], [12]. Nonetheless, most okara is treated at high temperatures, resulting in denaturation and decreased functionality [13]. Recent research has shown that proteins extracted in extremely alkaline environments have enhanced functional properties [14]. The assistance of physical methods can further improve the extraction efficiency and functional properties of proteins. Microwave treatment [15], high pressure [16], ultrasonic treatment [17], and steaming [18] are used to extract proteins with improved functional properties. Ultrasound pretreatment can improve the extraction efficiency of protein through the implosion of cavitation bubbles, the formation of microjets, disturbances in microporous particles, and the maximization of foaming stability [19], [20]. In our previous study, compared with traditional ultrasound-assisted alkali treatment, alternating ultrasound/alkali treatment at the same power and time can extract insoluble dietary fiber with greater functional properties [2]. In particular, the protein in an alkaline solution can be simultaneously recycled and utilized.

Therefore, this study focused on the advantages of alternating ultrasound/alkali extraction of OP over traditional (ultrasound-assisted) alkali treatment. It is highly anticipated that the non-continued cavitation and thermal energy output via alternating ultrasound/alkali treatments can effectively improve the structure and functional properties of OP, thus affecting their application prospects. The OP structure was analyzed by Fourier transform infrared spectroscopy (FTIR), fluorescence spectroscopy, and scanning electron microscopy (SEM); subsequently, the solubility, water/oil holding capacity, and emulsifying properties of the OP were tested. Finally, the 28-day storage stability of the OP-based emulsion was monitored to explore the enormous development potential of a stable emulsion based on OP extracted by alternating ultrasonic/alkali treatment.

## 2 Materials and methods

### 2.1 Materials

Okara was collected from a local market (Shandong, China). Sodium hydroxide and hydrochloric acid were purchased from Beijing Tong Guang Fine Chemicals Co., Ltd. (Beijing, China). Sodium dodecyl sulfate (SDS) (90 %) and 8-anilino-1-naphthalenesulfonic acid (ANS) (98 %) were purchased from Shanghai Yuanye Biotechnology Co., Ltd. (Shanghai, China). Commercial soybean oil was purchased from Yihai Kerry Arawana Holdings Co., Ltd. (Shanghai, China). All other chemicals used in this experiment were of analytical grade, and the solutions were prepared with deionized water.

### 2.2 Extraction of okara protein

**Fig. 1:**
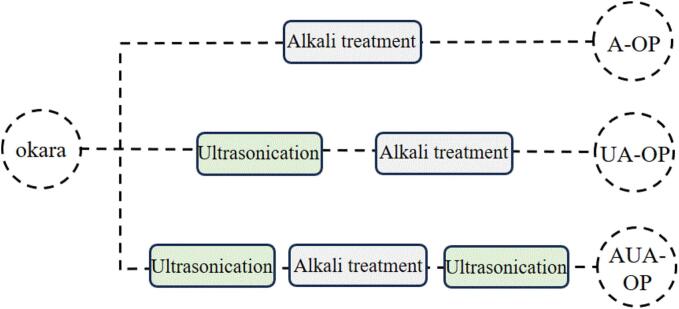
Preparation methods of A-OP, UA-OP, and AUA-OP.

The extraction method for okara protein was described in our previous study [2]. First, the collected okara was washed, crushed, dried, sieved, and then defatted with n-hexane, and the fat, protein, dietary fiber, and moisture contents of the collected okara were calculated as 1.08 %, 23.50 %, 68.15 % and 2.69 %, respectively. The obtained powdered okara (1 g) was mixed with a hybrid alkali mixture (30 mL) at pH 12.0 for 2 h to extract okara protein (A-OP). Moreover, ultrasonication at 400 W for 10 min was conducted before alkali extraction to prepare ultrasonic/alkali-treated okara protein (UA-OP). For alternating ultrasonic/alkali-treated okara protein (AUA-OP), ultrasonication at 400 W of ultrasonic power for 5 min was conducted before and after alkali extraction. The schematic diagram of the three preparation methods is clearly illustrated in Fig. 1. Then, the three clarified alkali extraction solutions after centrifugation were subsequently collected and adjusted to pH 4.5 to obtain precipitates. After several washes, these precipitates were adjusted to pH 7.0, freeze-dried, and stored. The protein purity of the prepared A-OP, UA-OP, and AUA-OP samples was measured via a Dumas nitrogen analyzer (NDA 701, Velp, Italy) with a nitrogen conversion factor of 6.25. The yield of each sample was calculated as follows:

(1) $$\mathrm{Yield}\;\left(\%\right)\;=M/M_{0}\;\times 100\%$$

where M_0_ (g) is the original weight of okara before alkali/ultrasonic treatment and M (g) is the weight of the extracted OP.

### 2.3 Emulsion preparation

In accordance with the methods of Tao et al. [18], 0.01 g/mL of the prepared OP solution (9 mL) and soybean oil (1 mL) were homogenized by an ultrasonic cell disrupter (400 W, 5 min) in an ice bath. The resulting O/W emulsion was stored at room temperature for 28 days to evaluate the storage stability of the samples.

### 2.4 Structure characterization

#### 2.4.1 Fourier transform infrared spectroscopy (FTIR) analysis

The FTIR spectra of the prepared samples were measured via dried KBr tableting using an FTIR spectrometer (Tensor 27, Bruker, Germany), and the transmittance of the samples was recorded from 500 to 4000  cm^−1^ during 64 scans.

#### 2.4.2 Fluorescence spectroscopy analysis

The prepared OP (1 mg) was mixed with a PBS solution (0.01 M, pH 7.4, 50 mL), and a fluorescence spectrophotometer (F97, Lengguang, China) was used for analysis, in which the excitation wavelength was 290 nm, the scanning wavelength ranged from 300 to 500 nm, and both the excitation slit and the emission slit were 5 nm.

#### 2.4.3 Surface hydrophobicity (H_0_)

The H_0_ of the prepared samples was measured following the method reported by Wu et al. [21]. The OP solution of a certain concentration calculated by the Bradford assay mentioned above was diluted into different concentration gradients (0.05 mg/mL, 0.1 mg/mL, 0.2 mg/mL, 0.3 mg/mL, 0.4 mg/mL, and 0.5 mg/mL). Then, as a fluorescent probe, ANS (0.1 mL) was reacted with protein solutions (10 mL) of different concentrations in the dark for 10 min, and a fluorescence spectrophotometer was used to record the relative fluorescence intensity of the samples. The emission wavelength was 484 nm, and the excitation wavelength was 365 nm. The H_0_ value was determined by calculating the initial slope of the relative fluorescence intensity and protein concentration.

#### 2.4.4 Sodium dodecyl sulfate–polyacrylamide gel electrophoresis (SDS-PAGE)

In accordance with the methods of Laemmli [22] with some modifications, 40 mg of soy protein isolates (SPI), A-OP, UA-OP, or AUA-OP were dissolved in 2 mL of protein mixture (20 mM Tris-HCl pH 8.0, 200 mM NaCl, 1 mM EDTA, or 0.5 % NP40), respectively. The prepared samples (8 μL) were mixed with 5 × loading buffer (10 mM Tris-HCl, pH 8.0; 2 % SDS; 10 % glycerol; 5 % 2-mercaptoethanol; and 0.02 % bromophenol blue) in boiling water for 3 min and then centrifuged for 5 min before loading. The supernatant (5 μL) was loaded onto the gel (12 % separation gel and 6 % stacking gel) for electrophoresis at 160 V. After 1 h, the completed protein gel was stained with Coomassie brilliant blue for 10 min and subsequently photographed and observed after decolorization.

#### 2.4.5 Scanning electron microscopy (SEM) analysis

The microstructures of the different samples were examined using a scanning electron microscope (SU8010, Hitachi, Japan). The prepared samples were pasted onto adhesive carbon and then coated with gold; subsequently, images were acquired at an accelerating voltage of 10 kV.

### 2.5 ζ-potential and size

To measure the ζ-potential and Z-average of the samples, the prepared OP (1 mg) was mixed with PBS solution (5 mL) to obtain an evenly dispersed protein suspension, which was determined at room temperature using a zeta-potential analyzer (Zetasizer Nano Zs, Malvern, England), and the polymer dispersity index (PDI) was simultaneously recorded.

### 2.6 Functional properties

#### 2.6.1 Solubility

The prepared OP (1 g) mixed with distilled water (100 mL) was stirred at room temperature for 1 h and then centrifuged at 10,000  rpm for 10  min to separate the protein supernatant. The protein content was measured via the Bradford assay [23]. In brief, the resulting supernatant (50 μL) was mixed with Coomassie blue staining solution (3 mL) for 10 min. Next, the mixture was detected by a microplate reader (Spectra MAX190, Molecular Devices, USA) at 595 nm with deionized water as a blank control and 0.524 g/L protein solution (bovine serum albumin) as a standard. The protein content was calculated via the following equation:

(2) $$\mathrm{Protein}\;solubility\;\left(g/g\right)\;=\;\left(A_{m}-\;A_{b}\right)/\left(A_{s}-\;A_{b}\right)\times C\times V/M$$

where A_m_, A_s_, and A_b_ represent the absorbances of the samples, protein standard solution, and blank, respectively. C (g/L) is the concentration of protein standard solution, V (L) is the total volume of OP supernatant, and M (g) is the weight of the OP.

#### 2.6.2 Water/oil holding capacity (WHC/OHC)

50 mg of OP was mixed with 1 mL of deionized water or soybean oil for 2 h at room temperature. Next, the mixture was centrifuged at 8000 rpm for 20 min, and the water or oil-loaded OP was separated and weighed. The WHC and OHC were calculated by the following equations [9]:

(3) $$\mathrm{WHC}\;\left(g/g\right)\;=\left(M_{w}-M\right)/M$$

(4) $$\mathrm{OHC}\;\left(g/g\right)\;=\left(M_{O}-M\right)/M$$

where M (g) is the weight of the sample before adding water/oil and where M_w_ (g) and M_O_ (g) are the weights of the sediment after centrifugation.

#### 2.6.3 Viscosity

A rheometer (Physical MCR 502, Anton Paar, Austria) was used to measure the viscosity of different OP-based emulsions. The prepared emulsion (0.75 mL) was sheared in dynamic frequency scanning mode at room temperature. The scanning frequency range was set as 0.1–100 rad/s, and the strain amplitude was 1 % [24].

#### 2.6.4 Emulsifying properties

Soybean oil (1 mL) and 10 mg/mL OP suspension (9 mL) were homogenized under 400 W ultrasonication for 3 min. After 0 min and 10 min, the emulsion (50 μL) at the bottom was transferred into a 1 mg/mL SDS solution (100 mL), and then the absorbance of the samples was recorded via a microplate reader at 500 nm with an SDS solution (1 mg/mL) as a blank control. The EAI and ESI were calculated as follows [25]:

(5) $$\mathrm{EAI}\;\left(m^{2}/g\right)\;=\;\left(2\;\times \;2.303\times A_{0}\times N\right)/\left(\varphi \times C\times L\times 10000\right)$$

(6) $$\mathrm{ESI}\;\left(min\right)\;=\;A_{0}/\left(A_{0}-\;A_{10}\right)\times 10$$

where A_0_ and A_10_ are the absorbances of the samples at 0 min and 10 min, respectively; N is the dilution ratio; φ is the volume fraction of the oil phase; C (g/mL) is the concentration of the OP suspension; and L is the optical path length of the colorimetric dish.

### 2.7 Emulsion stability

To evaluate the storage stability of the prepared emulsion, the samples were collected on days 1, 7, 14, and 28 to observe the changes in optical image, micromorphology, droplet size, and creaming index (CI). According to the method described by Tang and Huang [26], the micromorphology of the emulsions was obtained using an optical microscope (CX23, Olympus, Japan) with 10-fold eyepiece and 40-fold objective lenses, and the average diameter of the emulsion droplets in the obtained images was measured by Nano measurer software. Moreover, the CI was calculated according to the following equation:

(7) $$\mathrm{CI}\;\left(\%\right)\;=H/H_{a}\;\times 100\%$$

where H is the height of the serum layer at the bottom from the emulsions, and H_a_ is the total height of the serum and emulsion mixture.

### 2.8 Statistical analyses

All the quantitative experiments were conducted in triplicate, and the results were analyzed using Origin 2018 and SPSS 22.0 statistical software. Statistical significance was determined at a level of *P* < 0.05 using Duncan’s multiple range test.

## 3 Results and discussion

### 3.1 Yield and purity

**Fig. 2:**
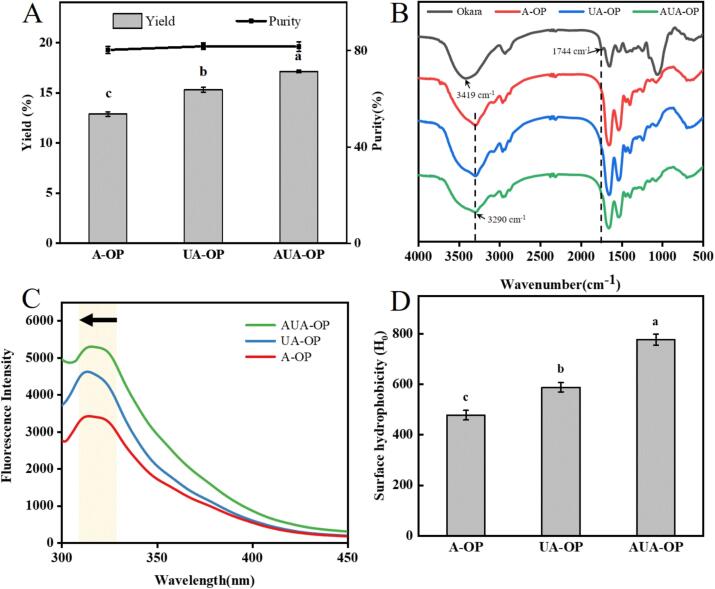
Yield and purity (A), FTIR spectra (B), fluorescence spectra (C), and surface hydrophobicity (D) of A-OP, UA-OP, and AUA-OP.

The protein in okara treated with different methods was enriched by pH-driven precipitation, and the yields of the different obtained OP are shown in Fig. 2A. The protein content of okara was calculated as 23.5 %. After different treatments, the yields of A-OP, UA-OP, and AUA-OP were 12.88 %, 15.30 %, and 17.13 %, respectively. Ultrasound disrupted the okara matrix, released tightly stacked cellular components, and even effectively separated carbohydrate-protein interactions via noncovalent binding, thus increasing the yield of OP [27]. Notably, the yield of AUA-OP was the highest, which might be attributed to the fact that alternating ultrasound promoted more effective penetration of solvents into the okara interior [15]. However, no significant changes in protein purity were detected among the different samples, and the protein content of all the samples was calculated to be about 80 % (Fig. 2A), which was similar to the results of previous optimized technique studies on OP extraction [28], [29].

### 3.2 Structure analysis

#### 3.2.1 FTIR analysis

The FTIR spectra of A-OP, UA-OP, and AUA-OP are plotted in Fig. 2B. The broad absorption peak at about 3290 cm^−1^ is assigned to O–H stretching, and the asymmetric and symmetric stretching of C-H were found at 2929 cm^−1^ and 2869 cm^−1^, respectively [9], [20]. The characteristic absorption peaks of amide I, amide II, and amide III from proteins are located at 1600–1700 cm^−1^, 1500–1600 cm^−1^, and 1240–1400 cm^−1^, respectively [25]. Compared with that of okara, the peak located at 1744 cm^−1^, corresponding to the stretching vibrations of C = O from the aldehyde group in lignin, disappears in the FTIR spectrum of the extracted OP, suggesting the removal of impurities [2]. In addition, an obvious blueshift from 3419 cm^−1^ to 3290 cm^−1^ was observed due to increased hydrogen bonding, which could be further explained by the secondary structure of the protein [30]. As shown in Table 1, AUA-OP resulted in a greater α-helix content and lower β-sheet content than A-OP and UA-OP did, implying increased hydrogen bonding interactions between protein molecules, indicating that alternating ultrasonic/alkali treatment improved the structure of the extracted protein to be more stable [31]. Moreover, a lower β-sheet content usually reflects greater surface hydrophobicity [12]. Notably, the β-turn content of AUA-OP significantly decreased, indicating a reduction in the degree of protein aggregation, which caused a decrease in particle size [21]. After ultrasound-assisted processing, the random coil content of UA-OP and AUA-OP significantly decreased, confirming the positive effect of ultrasound on the transformation of proteins into stable structures.

**Table 1:** Effects of pretreatments on the secondary structure percentage of A-OP, UA-OP, and AUA-OP.

	A-OP (%)	UA-OP (%)	AUA-OP (%)
α-helix	32.37 ± 0.46^c^	35.53 ± 0.85^b^	43.10 ± 0.19^a^
β-sheet	29.07 ± 0.23^a^	27.19 ± 0.84^b^	21.98 ± 0.08^c^
β-turn	24.81 ± 0.54^a^	24.31 ± 1.16^a^	22.59 ± 0.25^b^
random coil	13.74 ± 0.13^a^	12.96 ± 0.18^b^	12.33 ± 0.14^c^

Note: values marked with different letters in the same rows indicate the significant different means between different groups statistically (*P* < 0.05).

#### 3.2.2 Fluorescence spectra analysis

The fluorescence spectra in Fig. 2C reveal the spatial conformation of the protein, and the maximum absorption peak (λ_max_) was attributed mainly to the tryptophan residue in the prepared OP [32]. The order of fluorescence intensity was AUA-OP > UA-OP > A-OP, indicating that ultrasonic cavitation led to more chromophores within the molecule and that alternating ultrasonic/alkali treatment further promoted the exposure of internal functional groups. Compared with the λ_max_ of A-OP and UA-OP located at 315 nm, a significant redshift in the λ_max_ of AUA-OP was found at 321 nm, which was related to an increase in solvent polarity caused by more tryptophan residues in contact with the polar solvents [33]. It is suggested that the OP with more exposed functional groups could be effectively extracted by alternating ultrasonic/alkali treatment, which was consistent with the trend of changes in the secondary structure of the OP and beneficial for subsequent emulsification applications.

### 3.3 Protein H_0_

As a number of hydrophobic groups on the surface of protein molecules in contact with a polar aqueous environment, H_0_ is an important factor reflecting protein folding status and affects protein functional properties [34]. The AUA-OP treated by alternating ultrasonication clearly presented the highest H_0_ value, as shown in Fig. 2D, followed by UA-OP. The increased H_0_ value of the protein treated by ultrasonic pretreatment was also reported by Zhao et al. [34], whereas Tao et al. [18] reported that the H_0_ value of the protein decreased after ultrasonication. These changes in the H_0_ value of the protein were related mainly to the ultrasonication time and power, which affected the dissociation and reaggregation of the protein [33]. The greater hydrophobic interaction of AUA-OP might be attributed to the fact that during alternating ultrasonic/alkali treatment of okara, ultrasound preliminarily disintegrated the okara structure and induced protein unfolding, causing the hydrophobic groups in okara to be exposed. After alkaline hydrolysis, ultrasonication amplified the collapse effect, resulting in further enhancement of the number of hydrophobic groups in the system [2].

### 3.4 SDS-PAGE analysis

**Fig. 3:**
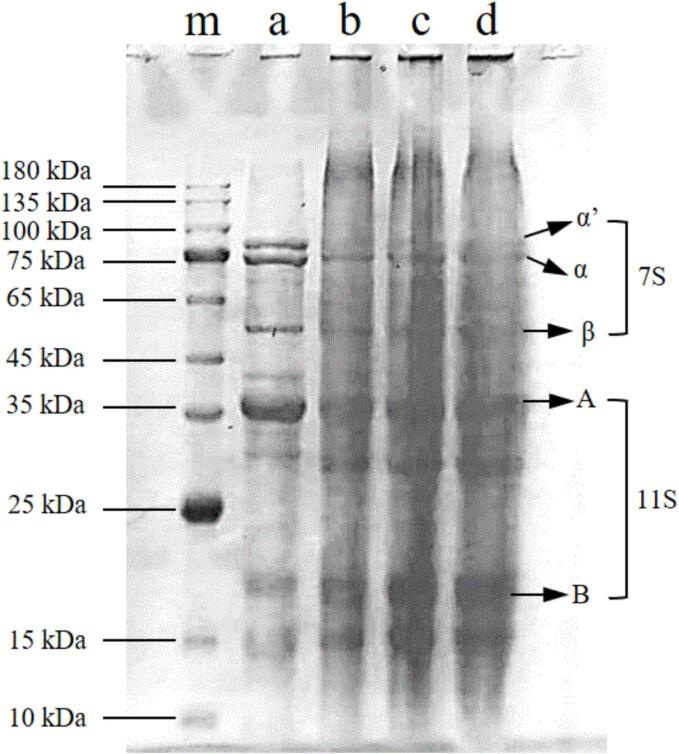
SDS-PAGE electrophoretic profiles of molecular weight marker (m), SPI (a), A-OP (b), UA-OP (c), and AUA-OP (d).

The SDS-PAGE patterns of the SPIs and extracted OP were obtained to explore the effects of ultrasonication on protein subunits (Fig. 3). All the samples presented protein subunits with molecular weights (M_w_) between 10 and 100 kDa. The main bands at about 87, 74, and 55 kDa correspond to the α’, α, and β subunits of β-conglycinin in the 7S protein, and acidic subunit A (∼36 kDa) and basic subunit B (∼18 kDa) appear in the 11S protein, which are the main components of the SPI [16]. These results were similar to previously reported SPI protein M_w_ values [16], [32]. Compared with those of SPI, the SDS-PAGE patterns of OP did not obviously change, indicating that ultrasound-assisted treatment did not affect the primary structure of the proteins, as reported by Jiang et al. [14] and Hu et al. [19].

### 3.5 Morphological characteristics

**Fig. 4:**
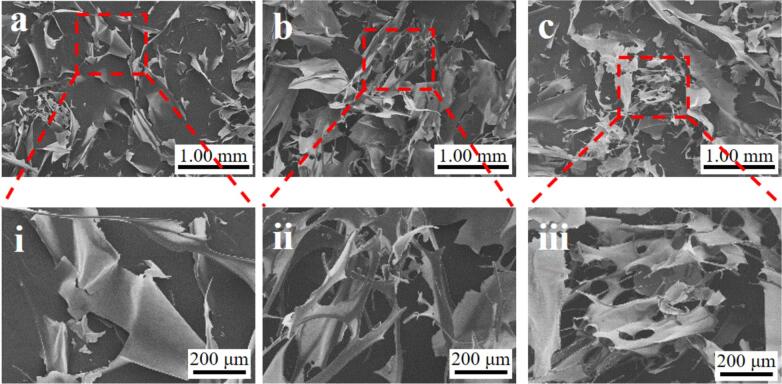
Morphology (×30) of A-OP (a), UA-OP (b), and AUA-OP (c); Morphology (×100) of A-OP (i), UA-OP (ii), and AUA-OP (iii).

The morphology of the OP extracted by different treatments was examined to observe the changes in microstructure. As shown in Fig. 4, the prepared A-OP mainly displayed a smooth sheet structure, whereas UA-OP and AUA-OP presented a porous sheet structure, which was attributed to the cavitation effect of ultrasonication [2]. Similar phenomena were also reported in black bean protein [33] and quinoa protein [35] treated with ultrasound. The large and smooth sheet-like structure was mainly due to the increased exposure of hydrophobic groups under extremely alkaline extraction conditions (pH 12.0) [15]. Importantly, the structure of AUA-OP was looser, and the fragment particles were smaller. The mechanical shearing forces generated by ultrasound opened the protein structure, causing molecular unfolding via peptide bond disruption, indicating that the reduction in β-sheets promoted structural looseness [36].

### 3.6 ζ-potential and size

The ζ-potential and particle size are shown in Table 2. In general, an absolute value of the ζ-potential higher than 20 mV indicates that a sample can be stably dispersed in the solvent, and a higher absolute value of the ζ-potential represents stronger electrostatic interactions between molecules [26]. As shown in this table, the OP particles dispersed in distilled water (pH > pl) carried negative charges, and the order of the absolute value of the ζ-potential was AUA-OP > UA-OP > A-OP, illustrating that the hydrophobic regions of proteins exposed via ultrasonic treatment led to an increase in surface negative charges, which caused stronger electrostatic repulsion between charges [13]. Moreover, the Z-averages of A-OP, UA-OP, and AUA-OP were 156.23 nm, 111.13 nm, and 89.21 nm, respectively. Ultrasonic shearing was found to significantly reduce the particle size of the protein, as reflected by the size distribution shown in Fig. 4. The polydispersity index (PDI), which indicates the uniformity of particle distribution, is also documented in Table 2. This table illustrates a more stable and uniformly dispersed system in the AUA-OP suspension. The increased dispersion stability and reduced particle size in AUA-OP can be attributed to the exposure, shearing, and loosening of structural proteins in okara due to ultrasonic pretreatment. Subsequent hydrolysis further promotes the degradation of proteins, enhancing the collapse effect [2].

**Table 2:** ζ-potential, Z-average, and PDI of A-OP, UA-OP, and AUA-OP.

Indicators	A-OP	UA-OP	AUA-OP
ζ-potential (mV)	−27.96 ± 2.04^a^	−34.48 ± 1.21^b^	−40.39 ± 0.74^c^
Z-average (nm)	156.23 ± 2.41^a^	111.13 ± 3.85^b^	89.21 ± 7.46^c^
PDI	0.65 ± 0.04^a^	0.44 ± 0.01^b^	0.34 ± 0.02^c^

Note: values marked with different letters in the same rows I ndicate the significant different means between different groups statistically (*P* < 0.05).

### 3.7 Functional properties

#### 3.7.1 Protein solubility

**Fig. 5:**
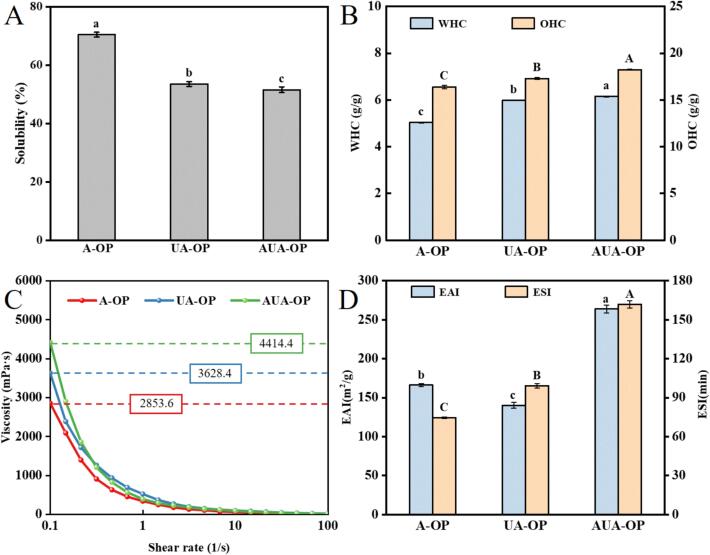
Solubility (A), WHC and OHC (B), viscosity (C), and EAI and ESI (D) of A-OP, UA-OP, and AUA-OP.

Solubility is a reliable indicator for determining protein hydration ability [19]. As shown in Fig. 5A, the solubility of the extracted protein was ranked as A-OP > UA-OP > AUA-OP. The decrease in solubility was related to changes in the hydrophobic structure of the proteins. As mentioned in the analysis of H_0_, ultrasound caused an increase in exposed hydrophobic groups, leading to large molecular aggregates driven by hydrophobic interactions between proteins and resulting in a decrease in solubility [33]. A similar phenomenon was also reported by Wang et al. [25].

#### 3.7.2 WHC and OHC

An improvement in the WHC and OHC usually enhances the texture, taste, and flavor of protein-based products [5]. The WHC and OHC of the prepared OP are shown in Fig. 5B. The WHC of A-OP, UA-OP, and AUA-OP were 5.03 g/g, 5.98 g/g, and 6.15 g/g, respectively, and the OHC of A-OP, UA-OP, and AUA-OP were 16.39 g/g, 17.29 g/g, and 18.23 g/g, respectively. Specifically, although the exposure of hydrophobic groups has a negative effect on the WHC of proteins, the induced dissociation of proteins during the ultrasound process can improve WHC [17]. Moreover, the increased WHC of OP might be ascribed to the decreased solubility and increased hydration areas exposed by unfolding. A previous study revealed that proteins with a higher H_0_ usually had a higher OHC, as more hydrophobic groups on the protein surface were involved in interactions with the fatty chains in the oil, thereby increasing the oil absorption capacity [37].

#### 3.7.3 Viscosity

In general, emulsions stabilized by smaller emulsified particles processes a higher viscosity [38]. Fig. 5C shows the shear viscosity scanning curves of emulsions stabilized with different OP. When the shear rate increased from 0.1 s^−1^ to 1 s^−1^, the viscosity of the prepared emulsions abruptly decreased; subsequently, when the shear rate gradually increased from 1 s^−1^ to 100 s^−1^, the viscosity of the emulsions tended to be smooth, showing typical shear thinning flow behavior, which is a common phenomenon in food-grade emulsions. The order of viscosity at 0.1 s^−1^ was A-OP emulsion < UA-OP emulsion < AUA-OP emulsion, suggesting that the smaller droplets of the emulsion stabilized by AUA-OP increased the contact surface area and interaction between particles, leading to an increase in emulsion viscosity, which supported emulsion stability.

#### 3.7.4 Emulsifying properties

The EAI and ESI are important indicators for evaluating the adsorption capacity of proteins at the oil–water interface (Fig. 5D). Compared with A-OP (166.12 m^2^/g), the EAI of UA-OP decreased (140.02 m^2^/g), whereas that of AUA-OP (263.77 m^2^/g) increased. The decrease in EAI might be attributed to protein denaturation caused by strong thermal energy under long-term ultrasound exposure, which affected the migration and absorption of proteins at the oil–water interface [14], [17]. Interestingly, the alternating ultrasonic/alkali treatment reduced the impact of sustained thermal energy on proteins under the same ultrasonic power and time, leading to an increase in the EAI. On the other hand, the ESIs of A-OP, UA-OP, and AUA-OP were 74.62 min, 99.18 min, and 161.94 min, respectively. The increase in the ESI was attributed mainly to protein molecules with smaller sizes and more stable structures promoting more rapid stabilization at the oil–water interface, thus increasing emulsion stability [25]. The high EAI and ESI of AUA-OP indicated that alternating ultrasonic/alkali treatment was a promising method for extracting proteins with strong emulsifying properties.

### 3.8 Emulsion stability

**Fig. 6:**
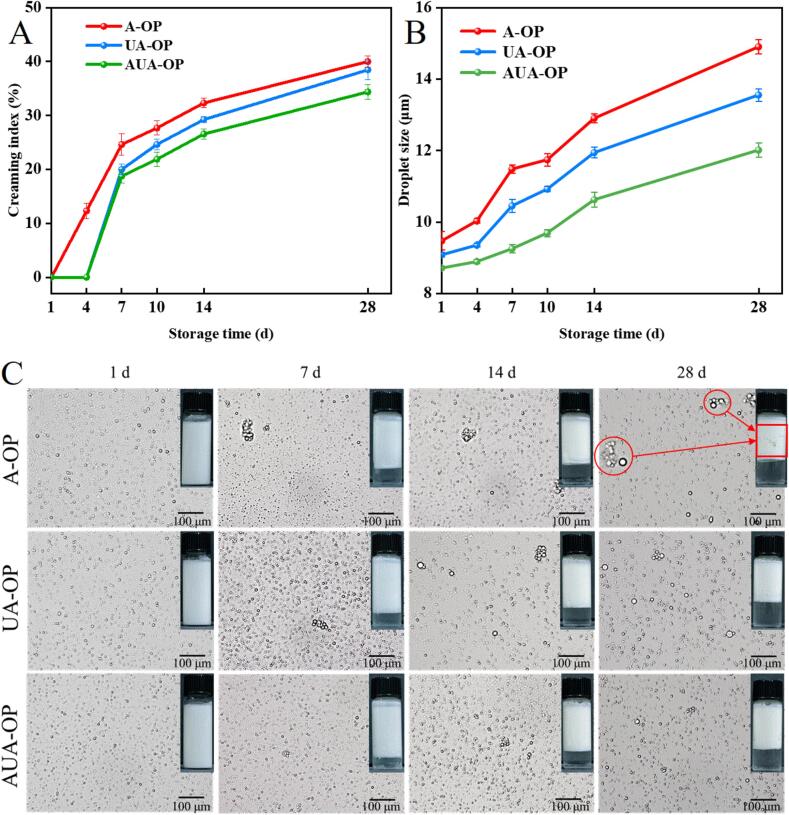
CI (A), average droplet size (B), and microscopy images (C) of emulsions stabilized by A-OP, UA-OP, and AUA-OP.

Flocculation and coalescence in emulsions typically occur during storage due to attractive interactions or aggregation of surfactants [11]. The stability of the prepared emulsion stabilized by the extracted OP was further studied, and optical images, droplet morphology, droplet size, and CI values of the different emulsions during 28 days of storage are shown in Fig. 6. CI is normally used to evaluate the stable equilibrium state between the oil phase and continuous phase, and a higher CI represents a lower physical stability of the emulsion [38]. After ultrasonication, the CI of the A-OP emulsion significantly decreased from 40 % to 38.46 % and further decreased to 34.38 % by alternating ultrasonic processing (Fig. 6A). This result indicated that unfolding of the protein structure was conducive to forming a denser emulsion network and effectively inhibited fat floating [39]. From a microscopic perspective (Fig. 6C), the A-OP emulsion showed obvious droplet aggregation on the 28th day, whereas the distribution of the OP emulsion treated with ultrasonication was more uniform. The average size of the droplets of different OP emulsions was subsequently measured (Fig. 6B), and the particle size ranking of the different emulsions on the 28th day was AUA-OP emulsion (14.90 μm) < UA-OP emulsion (13.55 μm) < A-OP emulsion (12.01 μm). Compared with the A-OP emulsion and UA-OP emulsion, the AUA-OP emulsion showed the lowest CI and smallest particle size. The stability mechanism of emulsions is related to their stable spatial structure and good electrostatic interactions [26]. AUA-OP, which has a small particle size, low PDI, and high dispersion stability, effectively overcame the aggregation of proteins at the oil–water interface. Furthermore, the diffusion and adsorption rates of particles at the oil/water interface are positively correlated with protein size [40]. These phenomena were consistent with the above analysis.

## 4 Conclusion

As mentioned above, the yield of OP extracted by alternating ultrasonic/alkaline treatment increased to 17.13 %, and no significant changes were found in the chemical structure. Compared with those of the A-OP and UA-OP emulsions, the AUA-OP-stabilized emulsion was more stable because of the greater surface hydrophobicity, greater electrostatic repulsion, lower particle size, and looser protein structure of AUA-OP, improving its functional characteristics, including higher water/oil holding capacity, ESI and EAI. This is due to the alternating ultrasonic/alkali treatment reduced the negative impact of prolonged-thermal energy on proteins, delaying protein denaturation. These advantages enhanced the storage stability and even showed good potential for extending the shelf-life of protein-based emulsions. These results provide new strategies for the extraction of various proteins and show attractive application prospects in the preparation of stable food-grade emulsions based on proteins.

## CRediT authorship contribution statement

**Lu Tang:** Writing – original draft, Conceptualization, Methodology, Formal analysis. **Xiaolin Liu:** Visualization, Investigation, Data curation. **Shiru Bai:** Resources. **Dan Zhao:** Visualization. **Xuzhen Guo:** Investigation. **Dandan Zhu:** Data curation. **Guiying Su:** Resources. **Bei Fan:** Validation, Supervision. **Bo Wang:** Resources, Methodology. **Liang Zhang:** Writing – review & editing, Project administration, Funding acquisition. **Fengzhong Wang:** Supervision, Project administration, Funding acquisition.

## Declaration of competing interest

The authors declare that they have no known competing financial interests or personal relationships that could have appeared to influence the work reported in this paper.
